# The impact of body mass and posterior adiposity indices in robotic adrenalectomy

**DOI:** 10.1007/s00464-025-12416-7

**Published:** 2025-11-25

**Authors:** Alexa Lisevick Kumar, Arantxa Sanchez, Kara Doffek, George Taylor, Tina W. F. Yen, Tracy S. Wang, Sophie Y. Dream

**Affiliations:** 1 Department of Surgery, Medical College of Wisconsin, Milwaukee, WI USA; 2 Department of Surgery, Clement J. Zablocki Veteran’s Affairs Hospital, Milwaukee, WI USA; 3 University of Alabama at Birmingham, 1808 7th Ave South, Birmingham, AL # 503U35294 USA

**Keywords:** Robotic adrenalectomy, Minimally invasive adrenalectomy, Body mass index, Posterior adiposity index, Adrenalectomy, Robotic surgery

## Abstract

**Background:**

Robotic adrenalectomy is performed via transabdominal (TA) or retroperitoneoscopic (RP) approaches, but the impact of body mass index (BMI) on robotic adrenalectomy outcomes in either approach is unclear. Posterior Adiposity Index (PAI), predictive of operative time in RP adrenalectomy, remains unevaluated with the robotic platform. This study assessed the impact of patient BMI and PAI on surgical outcomes in patients undergoing either TA or RP robotic adrenalectomy.

**Methods:**

This was a single-institution retrospective cohort study of adult patients who underwent robotic adrenalectomy from 5/1/2018 to 6/17/2024. Patients were analyzed by approach and BMI. The primary outcome was operative time. Secondary outcomes included estimated blood loss (EBL), length of stay (LOS), and complications within 30 days. The correlation of operative time to PAI, BMI, and patient characteristics was assessed.

**Results:**

149 patients (age 55 yrs. [IQR:42,66], 70% female) underwent 98 TA and 51 RP robotic adrenalectomies. Patients who underwent TA robotic adrenalectomies had larger nodules, higher BMI, and greater EBL; operative time, LOS, and complications were similar. Patients with elevated BMI (BMI ≥ 25) had similar preoperative characteristics to those with normal BMI (BMI < 25); they had longer operative times (129 min [107, 156] vs. 118 [90, 134], *p* = 0.045) and LOS (1 [1,1] vs 1 [1,1] *p* = 0.015) including two patients with 8-day LOS, but similar EBL (5 [5,10] vs. 10 [5,23], *p* = 0.828) and complication rates (15% vs 5%, *p* = 0.477). Among TA, but not RP, patients, BMI weakly correlated with operative time ($\rho$ = 0.320, p < 0.005). PAI did not correlate with operative time.

**Conclusion:**

This retrospective cohort study evaluating the impact of BMI and PAI on surgical outcomes in patients undergoing robotic adrenalectomy demonstrated that while elevated BMI (BMI ≥ 25) appears to prolong TA robotic adrenalectomy, PAI is not predictive of operative time in RP robotic adrenalectomy.

**Supplementary Information:**

The online version contains supplementary material available at 10.1007/s00464-025-12416-7.

Minimally invasive adrenalectomy is the favored surgical approach for managing benign adrenal pathologies as it reduces postoperative length of stay and postoperative pain [1–4]. There are two minimally invasive approaches: transabdominal (TA) and posterior retroperitoneoscopic (RP), both of which can be performed with robotic assistance. The TA approach provides familiar anatomy and patient positioning, whereas the RP approach avoids abdominal adhesions and may reduce the risk of postoperative incisional hernia [5]. Robotic assistance may improve surgeon ergonomics and facilitate dissection in larger tumors. While multiple studies have compared the safety and efficacy of TA and RP approaches, [6–9], only two have included the robotic platform [10, 11]. In one study, operative times, length of stay (LOS), and postoperative complications were similar between approaches [10], whereas in the other study, increasing body mass index (BMI) and the TA approach were associated with longer operative time [11]. Hence, the impact of BMI in robotic adrenalectomy remains unclear.

The Posterior Adiposity Index (PAI) was introduced by Lindeman et al*.* in 2018, as a novel preoperative quantitative measure to aid surgeons in determining the efficiency of the RP approach, after two prior studies had highlighted the challenges of pursuing the RP approach in obese patients [12, 13]. The authors defined PAI as the sum of the distances from the tip of the twelfth rib to Gerota’s fascia and the distance between Gerota’s fascia and kidney parenchyma. Cumulatively, these are the distances traversed in initial port placement and the dissection and creation of the retroperitoneal working space. The authors found a PAI ≥ 9 to be predictive of longer operative times in RP adrenalectomy, which may guide the surgeon to pursue the TA approach instead [14]. While these findings have been replicated in other studies, PAI remains unstudied in patients undergoing robotic adrenalectomy [15, 16]. The robotic platform is proposed to be beneficial for improving surgeon ergonomics and facilitating the dissection of larger tumors and for facilitating minimally invasive surgery in obese patients by supporting instrument articulation and anatomical visualization, which can present a challenge in traditional laparoscopy [17–19].

Given the expanded use of the robotic platform and gaps in the current literature to support the preoperative decision-making of surgeons choosing between TA and RP adrenalectomy, the current study was designed to explore the impact of BMI and PAI in robotic adrenalectomy. We sought to compare operative time, estimated blood loss (EBL), LOS, and complications by BMI in patients who underwent either TA robotic adrenalectomy or RP robotic adrenalectomy, and further, to analyze the predictiveness of PAI on robotic RP adrenalectomy outcomes.

## Materials and methods

This was a single-institution retrospective cohort study that included all patients who underwent unilateral robotic adrenalectomy for any surgical indication from May 1, 2018, to June 17, 2024. It utilized an institutional clinical outcomes adrenalectomy database composed of prospectively collected patient data extracted from the electronic health record (EHR). To minimize confounding factors, patients who underwent combined surgical procedures or cortical-sparing adrenalectomy were excluded. After exclusions, patients were divided into two cohorts by surgical approach: those who underwent TA adrenalectomy and those who underwent RP adrenalectomy. Institutional Review Board approval was received from the Medical College of Wisconsin Internal Review Board. Study results are reported in accordance with the STROBE statement.

Robotic TA adrenalectomy was performed with patients in a lateral decubitus position with slight backward rotation. The abdominal entry for TA adrenalectomy was generally performed at the anterior axillary line about 5 cm from the costal margin; typically, four working robotic port sites were used. Robotic RP adrenalectomy was performed with the patients in a genu-pectoral position using the Cloward saddle, with the patient prone and their hips and knees flexed at 90 degrees. A 12 mm port was placed an inch below the tip of the twelfth rib. Next, two additional 8 mm long robot-working ports were placed, one along the paraspinal muscles and the other lateral to the 12 mm port. For both robotic TA and RP approaches, a 5 mm assist port was utilized as needed. The procedures were performed using the Intuitive Surgical Xi system (Intuitive Surgical, Sunnyvale, CA, USA).

Surgical approach was left to the surgeon’s discretion and guided by multiple factors including operative indication, pulmonary comorbidities, abdominal surgical history, adrenal nodule diameter, indeterminate nodule appearance or concern for malignancy, and anatomical location of the adrenal gland in relation to proximal vasculature (renal, splenic) and organs (colon, kidney).

For all patients, demographic variables, preoperative comorbidities including diabetes mellitus (DM), asthma, chronic obstructive pulmonary disease (COPD), obstructive sleep apnea (OSA), and continuous positive airway pressure machine (CPAP) use, BMI (kg/m^2^), abdominal surgical history, American Society of Anesthesiologists Physical Status Classification (ASA), adrenal nodule diameter (cm) and laterality, and surgical indication were collected. Pelvic surgeries (i.e., cesarean section, total abdominal hysterectomy) were not considered as abdominal surgeries. For patients with multiple adrenal nodules on the side of surgical resection, the size of the largest nodule was considered. Surgical indications included ACTH-independent hypercortisolism, primary aldosteronism, mixed hypercortisolism and primary aldosteronism, pheochromocytoma, indeterminate lesions, adrenal nodule size, and metastases to the adrenal gland. Among patients who underwent RP adrenalectomy, the correlation of PAI to operative time and EBL was assessed. The PAI was calculated as the sum of the distance between the twelfth rib tip to Gerota’s fascia and the distance between Gerota’s fascia and kidney parenchyma for each patient individually by review by first author (A.L.) of their computed tomography (CT) scan obtained closest to the date of surgery. If a preoperative CT scan was not available or the relevant anatomy, such as the twelfth rib tip, was not visible on CT scan, the patient was excluded from relevant analyses.

Surgical outcomes were compared by surgical approach and by BMI. The primary outcome was operative time, defined as the time (minutes) from incision to closure as documented in the anesthesiology report in the EHR. Secondary analyses assessing the relationship of individual preoperative patient characteristics and operative time were completed. The secondary outcomes were EBL, LOS, and complication rates. EBL was defined as the blood loss (mL) as reported in the attending surgeon's operative note, trainee brief operative note, or anesthesiology report. If multiple reported EBLs were available, EBL as reported in the surgeon's operative note was utilized. If EBL was reported as a range, the median of the two reported volumes was utilized. LOS was defined as the time (days) from the start of the operation to discharge from the hospital. Surgical complications (including fascial dehiscence, wound infection, urinary tract infection (UTI), transfusions, pneumonia, pneumothorax, reoperation, readmission, and emergency department (ED) visit) within 30 days were identified via individual chart review.

### Statistical methods

Descriptive analyses of preoperative patient characteristics were completed and compared by surgical approach and by BMI. For BMI comparisons, patients who had a normal BMI (< 25 kg/m^2^) were compared to those who had an elevated BMI (≥ 25 kg/m^2^). Sub-analyses assessing outcomes by stratified BMI categories (< 25 kg/m^2^, 25–29.99 kg/m^2^, 30–34.99 kg/m^2^, ≥ 35 kg/m^2^) were completed. Continuous variables were reported as mean or median based on testing for normality using the Shapiro–Wilk normality test and categorical variables were reported as numbers and percentages. Continuous variables were assessed using Student’s *t*-tests, Mann–Whitney U-tests, and Kruskal–Wallis tests. Categorical variables were assessed using Fisher’s exact or chi-square tests. Spearman’s rank correlation was utilized to assess the relationship of PAI with operative time in patients that underwent RP adrenalectomy. The correlation of BMI to operative time, EBL, and LOS was assessed for TA and RP patients separately using Spearman’s rank correlation. For TA patients, we further assessed operative times by nodule size, as well as laterality and abdominal surgical history. For RP patients, we assessed operative times by laterality, pulmonary comorbidities, and preoperative CPAP use. Continuous variables were assessed using Spearman’s rank correlation whereas binary variables were assessed using Mann–Whitney U-tests. Clinical data points that were missing were attempted to be retrieved through manual chart review; where data remained unavailable, patients were excluded from those specific analyses and adjusted cohort numbers were reported. Statistical analyses were completed using Microsoft Excel and R version 4.4.1 by one author (A.L.K).

## Results

**Fig. 1:**
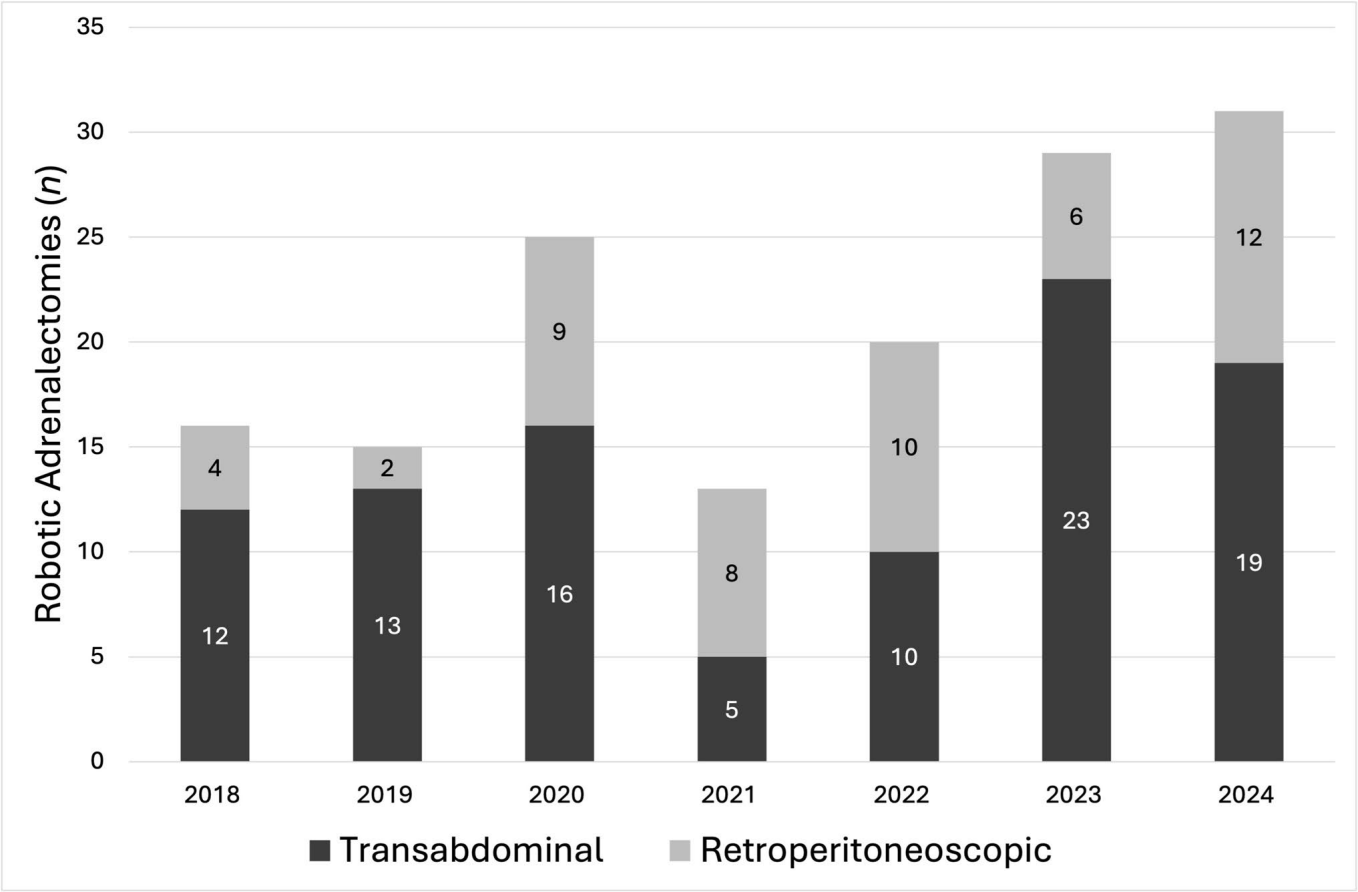
The volume and distribution by surgical approach of adrenalectomies over time included in this study

There were 157 patients who underwent robotic adrenalectomy during the study period; seven patients who underwent combined surgical procedures and one patient who underwent a cortical-sparing adrenalectomy were excluded. After exclusions, 98 patients who underwent TA adrenalectomy and 51 patients who underwent RP adrenalectomy were included in the final cohort. The first TA adrenalectomy was performed on 5/31/2018 and the first RP adrenalectomy was performed on 8/22/2018. There was variability annually in terms of the total number of adrenalectomies performed and the percentage by approach, with the TA approach being performed more often. (Fig. 1).

### Preoperative patient characteristics

The median age of the 149 patients was 55 years [IQR: 42, 66] and 70% were female. Patients who underwent TA adrenalectomy had larger nodule size and higher BMI than those who underwent RP adrenalectomy; otherwise, preoperative characteristics were similar between groups, as demonstrated in Table 1. The median BMI of all 149 patients was 32.7 kg/m^2^ [IQR: 27.7, 39.3] and 130 (87%) patients had elevated BMI. There was no difference between those with normal or elevated BMI with respect to preoperative prevalence of DM or pulmonary comorbidities (asthma, COPD, OSA), but patients with elevated BMI were more likely to use CPAP preoperatively. (Table 2).

**Table 1:** Preoperative patient characteristics by robotic adrenalectomy approach

	All patients (*n* = 149)	Transabdominal (*n* = 98)	Retroperitoneoscopic (*n* = 51)	*p*-value
Age, median [IQR]	55 [42, 66]	55.5 [41.3,66.8]	54 [43, 61]	0.596
Race				0.293
White/Caucasian	118	74	44	
Black/African American	23	18	5	
Asian/Pacific Islander	3	3	0	
Other	5	3	2	
Ethnicity				0.116
Hispanic/Latino/Spanish	4	1	3	
Sex				0.055
Female	104	74	30	
Male	45	24	21	
BMI, median, kg/m^2^ [IQR]	32.65 [27.7, 39.3]	33.93 [27.9, 42.1]	30.55 [27.5, 34.2]	0.011
BMI category				0.053
< 25 kg/m^2^	19	10	9	
25–29.99 kg/m^2^	36	22	14	
30–34.99 kg/m^2^	38	21	17	
35–39.99 kg/m^2^	20	15	5	
≥ 40 kg/m^2^	36	30	6	
History of prior abdominal surgery	56	36	20	0.906
# Prior abdominal surgeries, median [IQR]	0 [0,1]	0 [0,1]	0 [0,1]	0.586
ASA Physical Status Classification				0.203
ASA 2	16	11	5	
ASA 3	127	81	46	
ASA 4	6	6	0	
Nodule size, median, cm [IQR]	2.85 [1.7, 3.7] (*n* = 134)	3.2 [1.9, 3.8] (*n* = 91)	2.1 [1.4, 3.3] (*n* = 43)	0.025
Laterality				0.221
Left	79	56	23	
Right	70	42	28	
Indications				0.568
Adrenally mediated hypercortisolism	69	49	20	
Primary aldosteronism	43	24	19	
Mixed hypercortisolism/primary aldosteronism	7	4	3	
Pheochromocytoma	15	11	4	
Indeterminate lesion	5	3	2	
Size	6	5	1	
Metastases to adrenal	4	2	2	
Diabetes mellitus	47	34	13	0.336
Pulmonary comorbidities	88	45	16	0.124
CPAP use	25	19	6	0.3419

**Table 2:** Preoperative patient characteristics by patient BMI

	Normal BMI (BMI < 25) (*n* = 19)	Elevated BMI (BMI ≥ 25) (*n* = 130)	*p*-value
BMI, median [IQR]	23.5 [22.3, 24.1]	33.73 [29.5,41.3]	
Approach			0.301
Transabdominal	10	88	
Retroperitoneoscopic	9	42	
Sex			0.794
Female	14	90	
Male	5	40	
History of prior abdominal surgery	8	48	0.856
ASA Physical Status Classification			0.151
ASA 2	4	12	
ASA 3	14	113	
ASA 4	1	5	
Nodule size, median, cm [IQR]	2.35 [1.6, 4]	2.9 [1.8, 3.7]	0.865
Tumor laterality			0.834
Left	11	68	
Right	8	62	
Indications			0.232
Adrenally mediated hypercortisolism	10	59	
Primary aldosteronism	3	40	
Mixed hypercortisolism/primary aldosteronism	1	6	
Pheochromocytoma	5	10	
Indeterminate lesion	0	5	
Size	0	6	
Metastases to adrenal	0	4	
Diabetes mellitus	4	43	0.429
Pulmonary comorbidities	4	57	0.08
CPAP use	0	25	0.044

### Surgical outcomes: operative time, EBL, LOS, complications

Patients who underwent TA adrenalectomy had higher reported median EBL than those who underwent RP adrenalectomy; however, operative time, LOS, and complication rates were similar between groups, as shown in Table 3. Patients with elevated BMI had longer operative times, but similar EBL and complication rates to patients with normal BMI. (Table 3) Patients with BMI ≥ 35 kg/m^2^ appeared to have longer median operative times than patients with BMI < 25 kg/m^2^, 25–29.99 kg/m^2^, and 30–34.99 kg/m^2^ (*p* = 0.008), but EBL, LOS, and complication rates were similar. (Table 4) There were two patients with elevated BMI who had 8-day stays. One stay was prolonged related to a clotted arteriovenous fistula requiring percutaneous intervention, and the other stay was prolonged related to postoperative ileus and medical management of atrial fibrillation.

**Table 3:** Surgical outcomes by robotic adrenalectomy approach and by patient BMI

	All patients (*n* = 149)	TA (*n* = 98)	RP (*n* = 51)	*p*-value	Normal BMI (*n* = 19)	Elevated BMI (*n* = 130)	*p*-value
Operative time, median, mins [IQR]	127 [106,154] (*n* = 147)	131 [107,158] (*n* = 96)	122 [101, 142] (*n* = 51)	0.154	118 [90, 134] (*n* = 19)	129 [107, 156] (*n* = 128)	0.045
EBL, median, mL [IQR]	10 [5, 20] (*n* = 139)	10 [5, 20] (*n* = 93)	5 [5, 10] (*n* = 46)	< 0.005	10 [5, 23] (*n* = 19)	5 [5, 10] (*n* = 120)	0.828
LOS, median, days [IQR]	1 [1]	1 [1]	1 [1]	0.149	1 [1]	1 [1]	0.015
Complication, n (%)	21 (14%)	11 (11%)	10 (20%)	0.251	1 (5%)	20 (15%)	0.477

**Table 4:** Postoperative outcomes by BMI stratification

	< 25 kg/m^2^ (*n* = 19)	25–29.99 kg/m^2^ (*n* = 36)	30–34.99 kg/m^2^ (*n* = 38)	≥ 35 kg/m^2^ (*n* = 56)	*p*-value
Operative time, median, mins [IQR]	118 [105, 150]	121.5 [106, 154]	121 [108, 156]	137 [106, 155]	0.008
EBL, median, mL [IQR]	10 [5, 15]	10 [5, 20]	10 [5, 20]	10 [5, 20]	0.980
LOS, median, days [IQR]	1 [1]	1 [1]	1 [1]	1 [1]	0.5628
Postoperative complication, *n*	1 (5%)	5 (14%)	7 (18%)	8 (14%)	0.612

There were 21 patients (14%) who had complications within 30 days after surgery, as displayed in Table 5. Two patients required transfusions including one patient who underwent a TA adrenalectomy for pheochromocytoma (intraoperative and postoperative transfusions) and another patient who received a postoperative transfusion in the setting of retroperitoneal bleeding secondary to supratherapeutic anticoagulation requiring readmission within 30 days. Four patients who underwent RP adrenalectomy had a pneumothorax; all of which were identified on routine postoperative chest X-ray, were asymptomatic, and resolved on postoperative day one (POD1) without tube thoracostomy intervention. One patient underwent reoperation through a TA adrenalectomy approach without complication after their initial surgery was terminated given hemodynamic instability (secondary to inhalational anesthetic reaction) shortly after initiation of retroperitoneal dissection which did not improve with transition from prone to supine positioning.

**Table 5:** Complications within 30 days of robotic adrenalectomy

Complication	Patients, *n*
Emergency department visit	16
Hospital admission	6
Pneumothorax	4
Postoperative transfusion	2
UTI	2
Reoperation	1
Wound infection	1
Intraoperative transfusion	1
Pneumonia	0
Fascial dehiscence	0

In total, 16 patients presented to the ED within 30 days after surgery for various concerns including symptoms related to pancreatitis, UTI, fluid overload, seizure-like activity, superficial wound dehiscence at a TA adrenalectomy port site, abdominal pain following TA adrenalectomy, hypertensive urgency during postoperative medication titration for primary aldosteronism, unilateral extremity swelling, upper respiratory symptoms, chest pain following RP adrenalectomy, and a fall from standing. Six patients were admitted to the hospital within 30 days after surgery for various indications (workup of seizure activity, management of altered mental status, pain control, acute kidney injury).

### PAI correlation with operative time

Of the 51 patients who underwent RP adrenalectomy, the mean PAI was 6.7 (SD 2.1); 41 patients were found to have a PAI < 9 and 7 (13%) patients were found to have a PAI ≥ 9. For patients with a PAI > 9 the range was 9.2 to 11 with a median PAI of 10.1 [IQR: 10.0, 10.2]. Three patients were unable to have PAI calculated due to a lack of appropriate imaging. Patients with a PAI < 9, compared to those with PAI ≥ 9, had similar median operative time (122 min [IQR: 105, 144] vs 121 min [IQR: 95, 149], *p* = 0.793) and similar median EBL (5 mL [IQR: 2, 5] vs 5 mL [IQR: 5, 10], *p* = 0.306). There was no correlation between PAI and operative time ($\rho$ = 0.095, *p* = 0.515).

### Predictors of operative time in TA adrenalectomy

In patients who underwent TA adrenalectomy, BMI weakly positively correlated with operative time ($\rho \hspace{0.17em}$= 0.320, *p* < 0.005), but did not correlate with EBL ($\rho$ =-0.060, *p* = 0.568) or LOS ($\rho$ =0.104, *p* = 0.309). Nodule size did not correlate with operative time ($\rho \hspace{0.17em}$= − 0.048, *p* = 0.656). Laterality (left, 142 min [IQR: 108,174] vs. right, 125 min [IQR: 106,139], *p* = 0.078) and the presence of abdominal surgical history (yes, 123 min [IQR: 106, 165] vs. no, 135 [IQR: 109,153], *p* = 0.985) were not associated with operative time.

### Predictors of operative time in RP adrenalectomy

In patients who underwent RP adrenalectomy, BMI did not correlate with operative time ($\rho$ = 0.155, *p* = 0.277), EBL ($\rho$ = 0.050, *p* = 0.739), or LOS ($\rho$ = 0.111, *p* = 0.440). Laterality (left, 121 min [IQR: 95,133] vs. right, 123 min [IQR: 106, 154], *p* = 0.298), pulmonary comorbidities (yes, 135 min [IQR: 95, 162] vs. no, 121 min [IQR: 103, 133], *p* = 0.300), and preoperative CPAP use (yes, 144 min [IQR: 100,164] vs. 121 min [IQR: 101,135], *p* = 0.52) were not associated with operative time.

## Discussion

Minimally invasive adrenalectomy represents the gold standard for the management of most benign adrenal pathologies. While many studies have sought to describe patient factors that should guide surgical approach (TA vs. RP) in adrenalectomy, few have examined the role of BMI in robotic adrenalectomy, and none have examined PAI. Hence, this retrospective cohort study compared surgical outcomes, including operative time, EBL, LOS, and complications, in patients who underwent either TA or RP robotic adrenalectomy based on BMI and PAI.

In this study, most patients that underwent robotic adrenalectomy had elevated BMI, including 38% of the patients who had a BMI ≥ 35 kg/m^2^*,* and they had prolonged operative times compared to patients with normal BMI. In TA patients, but not RP patients, BMI correlated with operative time, suggesting that BMI may remain a meaningful preoperative consideration in determining TA robotic adrenalectomy efficiency. In contrast, BMI may be less useful in predicting operative efficiency of an RP approach when the robotic platform is used. The smaller impact of BMI in RP adrenalectomy may be due to the role of the robotic platform in improving the efficiency of the retroperitoneal dissection through instrument reach, visualization, and exposure. Isiktas et al*.* previously hypothesized that perioperative outcomes in severely obese patients (BMI ≥ 35 kg/m^2^) undergoing robotic adrenalectomy would be improved as compared to those undergoing non-robotic minimally invasive adrenalectomy, based on their reported experience of finding similar perioperative outcomes between obese patients undergoing robotic and laparoscopic adrenalectomy [20]. While their data failed to demonstrate improvement in RP robotic adrenalectomy outcomes, patients who underwent TA robotic adrenalectomy had shorter operative time and less EBL when the robotic platform was used [17]. Nonetheless, the observation in this study that patients with BMI > 35 kg/m^2^ had longer operative times than those with BMI < 25 kg/m^2^, BMI 25–29.99 kg/m^2^, or BMI 30–34.99 kg/m^2^ suggests that severe obesity may continue to prolong operative time, despite the robotic platform.

Ventilation safety and efficiency is a common concern raised when operating on patients with obesity in the prone position. In this study, patients with elevated BMI more often used CPAP preoperatively. Compared to patients with a normal BMI, patients with an elevated BMI (median BMI 33.73 kg/m^2^) did not have higher complication rates and RP patients with pulmonary comorbidities or preoperative CPAP use had similar operative times to those without. There was one RP patient who did develop hemodynamic instability shortly after beginning the retroperitoneal dissection, but this was unresolved with transitioning to supine positioning and determined to be related to an anesthetic reaction. Utilization of the Cloward saddle, which provides a concavity for the abdomen in prone positioning, may have been helpful in ventilating patients and could contribute to the similar surgical outcomes among patients with elevated BMI compared to normal BMI who underwent RP adrenalectomy. Prone positioning has been utilized among patients with obesity outside of the operating room, particularly in ICU settings, involving careful abdominal positioning supporting the chest and hips with pillows, as well as reverse Trendelenburg positioning, to avoid increasing abdominal pressure and organ compression [21]. Similarly, the Cloward saddle may aid in limiting compression of the retroperitoneum by the abdominal organs in morbidly obese patients in the prone position, which has also been highlighted as a concern in RP adrenalectomy [12]. For surgeons who are planning for an RP approach to improve intraoperative efficiency in patients with elevated BMI, patients can be counseled that the Cloward saddle can be used to facilitate safe ventilation while they are prone in surgery.

Comparing surgical approaches, the only difference in surgical outcomes was identified in patients who underwent TA adrenalectomy; they had higher EBL as compared to those who underwent RP adrenalectomy (median EBL of 10 mL versus 5 mL). A similar study comparing patients who underwent robotic adrenalectomy reported comparable EBL between those who underwent TA adrenalectomy versus RP adrenalectomy (10 mL vs. 5 mL) [10]. While 5 mL is a statistically significant difference, it is not a clinically meaningful difference, and retrospective quantification of EBL is inherently challenging and can be imprecise [22, 23].

To our knowledge this is the first study to evaluate the impact of PAI in robotic adrenalectomy. In this study, we aimed to apply the previously established PAI threshold of 9 for non-robotic RP adrenalectomy to a robotic adrenalectomy patient population. There was no difference in operative time in patients who underwent RP robotic adrenalectomy with a PAI of greater or less than 9. This threshold was previously identified by Lindeman et al. [14]. to be associated with prolonged operative times in non-robotic RP adrenalectomy. Pearlstein et al*.* found PAI to be associated with longer operative time on univariable analysis of non-robotic adrenalectomy patients; however, this effect did not persist in their multivariable regression model. Instead, they found periadrenal fat volume to be a superior predictor of increased operative time [15]. While their patient population’s PAI distribution (median, IQR) was similar to that of the current study, there was a greater proportion of patients in the current study with a BMI ≥ 40 (12% vs. 1.2%) who underwent RP adrenalectomy. In this study, we did not assess the impact of the thickness of perirenal fat tissue surrounding the adrenal gland as it has already been evaluated in robotic surgery. In a retrospective study of 112 patients who underwent robotic RP adrenalectomy, Gokceimam et al*.* found operative time was predicted by perirenal fat thickness (measured at the level of the renal hilum), with 18.5 mm as the critical threshold to predict an unfavorable operative time in robotic adrenalectomy [24]. There are other clinical factors that may influence operative approach that are considered by endocrine surgeons preoperatively, such as hypercortisolism or malignant disease. An important strength of the current study is that surgical indications were distributed similarly by surgical approach and by BMI cohort (normal vs. elevated).

This study is limited by several factors, including the measurement of EBL as previously stated. The selection of TA adrenalectomy versus RP adrenalectomy approach was not randomized and left to surgeon discretion*.* One strength of the current study is that it represents the outcomes of more than one operating surgeon and that each surgeon performs both surgical approaches. Nonetheless, the results of the study would be further strengthened by validation through a prospective, multi-institutional study inclusive of several junior and senior surgeons performing both approaches and randomization of patients to either approach. Importantly, most preoperative characteristics, including the distribution of patient body weight by BMI category, were similar when comparing patients by surgical approach. Finally, while univariable analyses were feasible to assess the impact of multiple binary and continuous preoperative characteristics on operative time in isolation, multivariable linear regression modeling of operative time was not possible given the skewed distribution of operative times. Future studies may benefit from the utilization of the recording technologies available through the robotic platform to characterize specific portions of the surgery itself, such as time elapsed in retroperitoneal dissection, vasculature ligation, or specimen extraction. Studies omitting time included for docking or skin closure, which tends to be variable but does not necessarily represent operative efficiency, may yield additional meaningful information regarding the operative efficiency of both approaches.

These findings provide evidence to support the decision-making of surgeons evaluating patients with elevated BMI for robotic adrenalectomy. For surgeons who perform both TA and RP approaches with similar expertise, it is important to consider that in patients with elevated BMI, an RP approach may be more efficient, given that pursuing a TA approach for these patients appears to prolong operative time despite the robotic platform. For surgeons who are considering an RP approach, calculation of PAI should be omitted when considering the robotic platform, as it has not been shown to be predictive in this setting.

## Supplementary Information

Below is the link to the electronic supplementary material.

Supplementary file1 (PNG 320 KB)
