# Supplementary figures and images for: The impact of body mass and posterior adiposity indices in robotic adrenalectomy
Source: Surg Endosc. 2025 Nov 25;40(2):1488–96. doi: 10.1007/s00464-025-12416-7 (PMC12881112; doi:10.1007/s00464-025-12416-7)

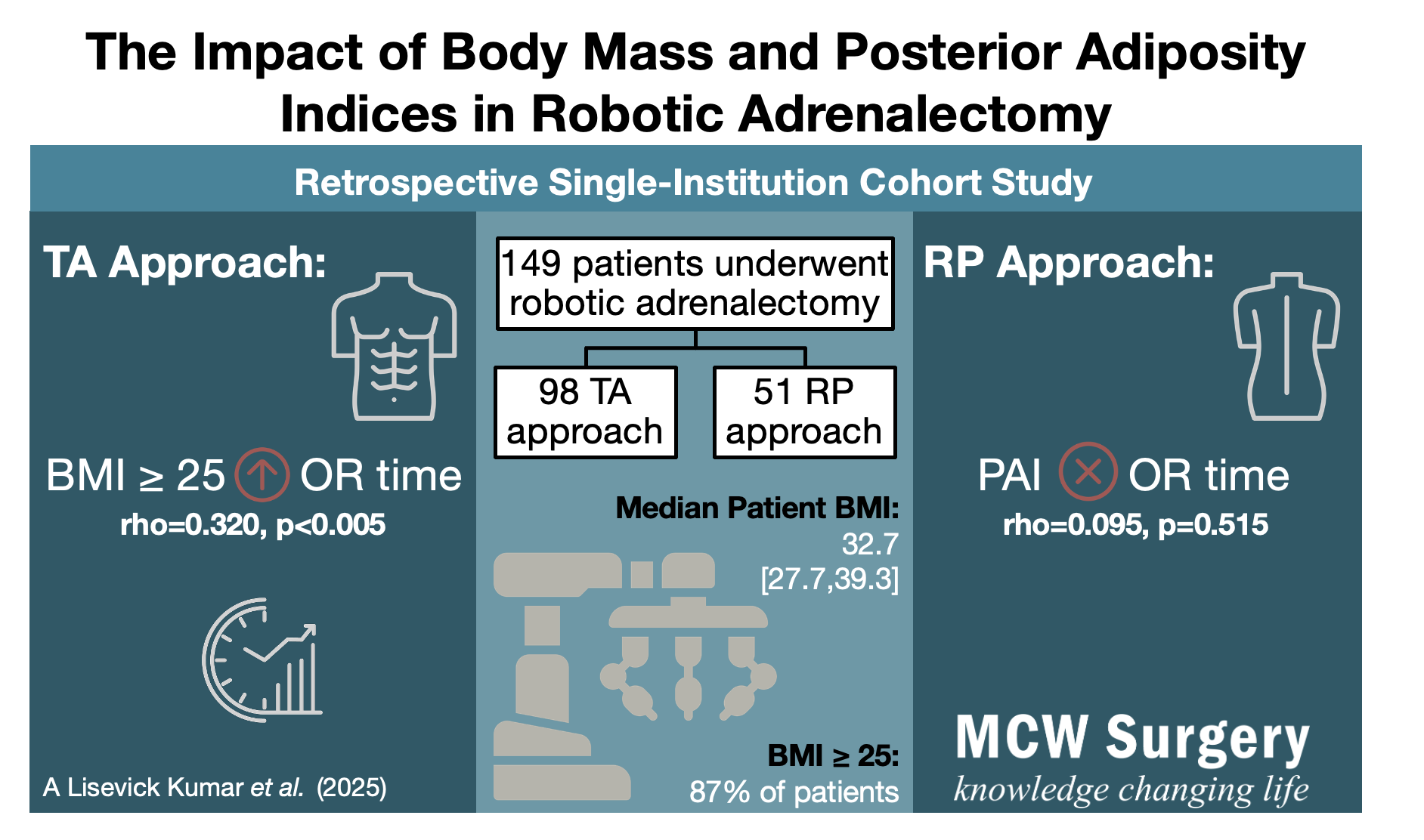

Supplement: Supplementary file 1 — Supplementary file1 (PNG 320 KB) [file 464_2025_12416_MOESM1_ESM.png]
